# Measuring strain in the exoskeleton of spiders—virtues and caveats

**DOI:** 10.1007/s00359-020-01458-y

**Published:** 2021-01-18

**Authors:** Reinhard Blickhan, Tom Weihmann, Friedrich G. Barth

**Affiliations:** 1 Science of Motion, Friedrich Schiller-University, Seidelstr. 20, 00749 Jena, Germany; 2 Institute of Zoology, University of Cologne, Zülpicher Str. 47b, 50674 Köln, Germany; 3 Department of Neuroscience and Developmental Biology, University of Vienna, Althanstr. 14, 1090 Wien, Austria

**Keywords:** Strain gauge, Mechanical sensitivity, Joint load, Locomotion, Sensory strain reception

## Abstract

The measurement of cuticular strain during locomotion using foil strain gauges provides information both on the loads of the exoskeleton bears and the adaptive value of the specific location of natural strain detectors (slit sense organs). Here, we critically review available literature. In tethered animals**,** by applying loads to the metatarsus tip**,** strain and mechanical sensitivity (*S* = strain/load) induced at various sites in the tibia were determined. The loci of the lyriform organs close to the tibia–metatarsus joint did not stand out by high strain. The strains induced at various sites during free locomotion can be interpreted based on *S* and, beyond the joint region, on beam theory. Spiders avoided laterad loading of the tibia–metatarsus joint during slow locomotion. Balancing body weight, joint flexors caused compressive strain at the posterior and dorsal tibia. While climbing upside down strain measurements indicate strong flexor activity. In future studies, a precise calculation and quantitative determination of strain at the sites of the lyriform organs will profit from more detailed data on the overall strain distribution, morphology, and material properties. The values and caveats of the strain gauge technology, the only one applicable to freely moving spiders, are discussed.

## Introduction

**Fig. 1:**
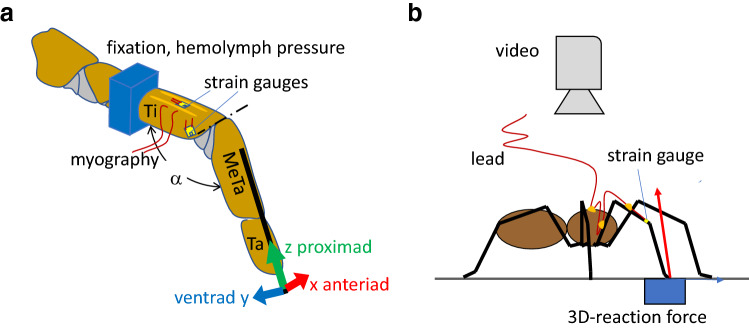
Setup of **a** tethered tarantula and **b** freely walking spiders. **a** Spiders were secured with tape on a brass holder; their second leg fixed with dental cement. At this site, the pressure at the dorsal hemolymph channel was registered. Trimmed strain gauges were affixed with cyanoacrylate. Here, we focus on two sites of gauge application: (1) the dorsal tibia and (2) the site of the lyriform organ HS8. Activity of the flexor *M. met. bilobatus* was registered. Using a needle fixed with beeswax to the metatarsus, the leg tip was deflected electromagnetically in three orthogonal directions. A custom-built force transducer allowed the registration of the force required to achieve the deflections. While moving the leg tip in the different directions against joint stiffness, the reaction force points in the same direction. However, flexion induced by muscles generates a dorsad, extension generated by hydraulic pressure a ventrad reaction force. *α*: dorso-ventral angle of the tibia–metatarsus joint; dash-dotted line: dorsal axis of the hinge joint. *Ti* tibia, *MeTa* metatarsus, *Ta* tarsus. Anteriad (*x*), ventrad (*y*), proximad (*z*) form a right-handed system of co-ordinates. **b** A strain gauge was bonded to the specified site at the tibia of the spider’s leg. Slack leads were guided across the leg to the prosoma and to a holder. In some cases, ground reaction force was measured in 3D with a custom-built force plate and kinematics registered with a video camera supported by mirrors (e.g., Barth 2002)

The exoskeleton of arthropods not only serves to support the muscle-skeletal system. In addition, via embedded sensors, it simultaneously gauges and transmits information about external and internal loads to the central nervous system. In spiders, behavioral studies in combination with the ablation of selected strain receptors illuminated the sensory mechanisms involved and the roles played in various behaviors (rev. Barth 2002, 2012; see also Barth 2020, a contribution to this SI). The influence of the arrangement of slits within compound-slit sense (lyriform) organs and the directional characteristics were investigated by measuring local deformation in plastic models (Barth et al. 1984; Barth and Pickelmann 1975; Barth et al. 1984), by applying the finite-element method (FEM; Hößl et al. 2006, 2007, 2009), and by interferometric in vivo measurement of the deformation of selected organs of *Cupiennius salei* (Schaber et al. 2012). Whereas much is known about the responses of slit sensilla to mechanical stimulation, investigations making use of the possibilities of modern technology to assess the deformation of whole skeletal segments or even structures such as entire legs under natural conditions are still missing. In a pioneering study, Barth et al. (1984) could show that the compound-slit sense organs at the distal end of the spider leg tibia (Fig. 1) are oriented roughly perpendicular to the principal stress trajectories to be expected during locomotion. Neither speckle photometry (e.g., Erf 2012) nor FEM (e.g., Yang 2019) has yet been applied to determine the strain distribution in the loaded tibia. They would be appropriate tools to investigate the mechanical implications of its local specializations (regarding the macro- and micro-structure, and the properties of the cuticular material including its local variation), and to identify representative loading conditions. The latter are of fundamental interest both with respect to an understanding of the adaptedness of the leg’s mechanical design and its relation to the mechanosensory feedback control of the spider’s motion. However, for the measurement of deformation and strain in live animals under natural conditions, there seems to be no practical alternative to the application of strain gauges.

We here critically review selected published results of strain monitoring in tethered tarantulas (Blickhan 1983; Blickhan and Barth 1985) and freely running *Cupiennius salei* (Brüssel 1987; Barth 2002). After describing the method of strain measurements with strain gauges, we document the strains induced by forces applied to the spider walking leg tip and thereby identify the mechanical sensitivity (*S* = strain/load) for different areas of the tibia, which is richly supplied with biological strain receptors (slit sensilla). In agreement with neurophysiological data (e.g., Barth 2002), the forces effectively stimulating the most intensively studied lyriform slit sense organ HS8 on the walking leg tibia are quantified. The mechanical sensitivity *S* of the dorsal tibia as documented in the literature is then used to predict the flexor activity during inverted climbing. Finally, we discuss the significance and potential drawbacks of the strain gauge technique for the measurement of arthropod skeletal strains.

## Materials and methods

The technology of measuring strains using strain gauges is well established in engineering and in biomechanics (e.g., Zdero et al. 2017). We here shortly outline the procedures of the strain gauge measurements previously applied and the corresponding setups used (Blickhan and Barth 1985; Barth 2002) to provide a sufficient basis for an understanding of its critical evaluation in the given context. The strain gauge technique is applicable to large spiders only and in experiments reviewed here the focus was on the tibia of the second walking leg. The diameter of the tibia of the selected species, hunting spiders *Cupiennius salei* (2–4 g; own stock; Barth 2002, 2020) and different species of tarantulas (18–26 g; *Aphonopelma* sp., *Brachypelma* sp., *Tapenauchcinus* sp.) ranged from 2 to 3.4 mm and the tibia length from 8.5 to 12 mm.

The core element of strain measurement was an ultra-miniature foil strain gauge (EA-13-008CL-120, Micro-Measurements, Raleigh, NC, United States). It was and still is the gauge with the smallest active area available, with thin wires (grid: 0.2 mm × 0.25 mm; constantan alloy; foil or wire thickness *h*_f_ ≈ 5 µm; Young’s modulus *E*_f_ = 1.6 10^11^ Pa; Technical Information Micro-Measurements), solder tabs (constantan alloy) for contacting, and an epoxid matrix (thickness *h*_b_ ≈ 25 µm; Young’s modulus *E*_b_ ≈ 3 10^9^ Pa). After trimming the gauge foil down to ca. 1 mm × 0.75 mm and contacting it with thin gold or copper wires (diameter < 70 µm), it was attached to various sites**,** especially at the spider’s tibia. A fast setting adhesive (CN, TML, Lithuania) was used for bonding. Secure adhesion required careful surface preparation including the removal of cuticular hairs, slight dissolution of the surface layer with ethanol, the surface roughening with scouring powder, and finally cleaning with ethanol. After adding a droplet of adhesive between foil and cuticle, a slight pressure (ca. 60 s) with a PTFE-protected finger or sponge provided secure adhesion. Foil strain gauges measure strain by change of the resistance of the thin wires due to the change in length. Correspondingly, only the strain in the direction of the wires is registered, orientation matters. The sensitivity of the material-dependent change in resistance (gauge factor), the supply voltage (as low as possible to avoid heating), and the amplification determined the signal amplitude in response to the experienced strain. Parallel resistors allowed for shunt calibration (System 2100, Vishay Micro-Measurements, Raleigh, USA). Measuring strains within the µε range (1 µε = 10^–6^ Δl/l; compression: strain < 0) required high amplification (typically × 60 000) and stable temperature. Due to curvature, for attachments perpendicular to the long axes of the tibia (HS8, tarantula), an increase of strain of 5% is expected ("Appendix 1", Eq. 1). Stiffening of the cuticle due to the gauge reduced strain by about 10%. Stiffening and the influence of a changing curvature (local buckling) are discussed in detail in the last chapter. The corresponding corrections were not applied to the values presented. Close to the tibia–metatarsus joint, the most extensively studied compound-slit organs are located (Barth and Libera 1970; Barth 2002, 2012, 2019; Hößl et al. 2009). In *Cupiennius salei* and in tarantulas, the joint is flexed by only four muscles, hemolymph pressure being the hydraulic antagonist. All spiders lack extensor muscles in this joint. In addition, the easy access to the joint facilitates the calculation of loads. The influence of inertia can be neglected during slow locomotion and high segmental loading is primarily caused by body weight. In a quasi-static approach, the vector of the joint load can be constructed knowing the ground reaction force, its point of application (center of pressure, COP), and the position of the tibia–metatarsus joint with respect to the COP (see below).

Experiments with tethered tarantulas (Fig. 1a) were used to estimate the strains introduced by loads induced in the directions (*x*-anteriad, *y*-ventrad, *z*-proximad) of the local metatarsus-fixed system of co-ordinates. Via an insect pin fixed to the long axis of the metatarsus, the latter was deflected (triangular time series, ca. ± 5 µm proximad, ca. ± 250 µm anteriad with respect to load-free posture; for precise deflection an electrodynamic vibrator V106, Ling and a PID controller according to Bohnenberger 1981 were used). The forces were measured with a custom-built force transducer (Blickhan and Barth 1985). Loads in the ventrad direction were generated by startling the animal with a brush. The forces were used to estimate the mechanical sensitivity (*S* = strain/force) in the different directions for various sites of the tibia. Thus, the interpretation of the strains observed in freely walking *Cupiennius salei* (Fig. 1b) and of the functional role of the slit sense organs located at the tibia was made possible. The setups for experiments on tethered and on free running animals were complemented by custom-built force transducers to measure specific loads applied to the metatarsus, by synthetic sensors to register local hemolymph pressure, and by 3D-force plates to register ground reaction forces during locomotion (Blickhan and Barth 1985, Brüssel 1987; Barth 2002).

Calculations within this manuscript were carried out with Matlab R14 (Mathworks, Natick, MA, USA).

## Load and strain in tethered spiders (tarantulas)

Experiments with tethered tarantulas were used to evaluate the influence of various loading parameters on local strains (set up: Fig. 1). The strain/force ratio serves as a quantitative measure of the mechanical sensitivity *S* at a particular location. We show that the site of the HS8 is sensitive to torsional load and to muscular flexion (Blickhan and Barth 1985) and line out how the hemolymph pressure must be considered. The mechanical sensitivities we then used to interpret the measurements on freely moving *Cupiennius salei*.

**Fig. 2:**
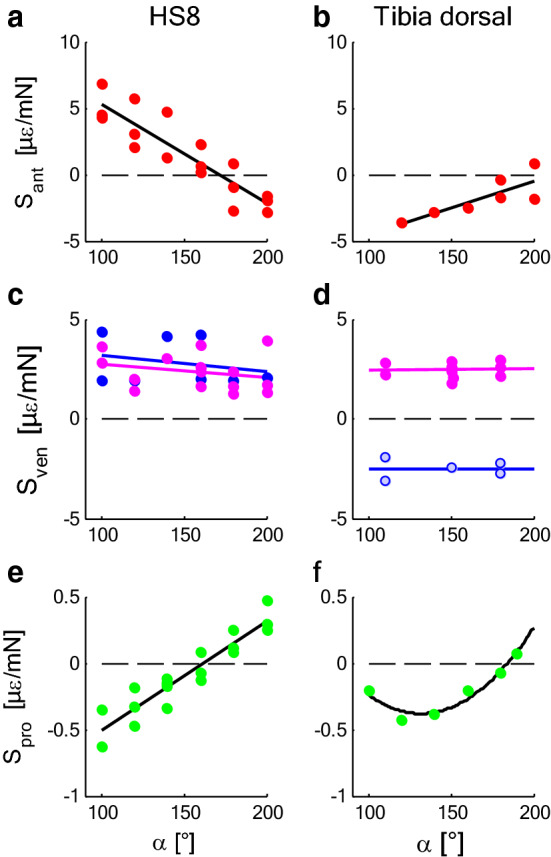
Mechanical sensitivity *S* in dependence of the joint angle *α* (s. Fig.1) at the site of the lyriform organ HS8 (left) and the dorsal tibia (right) of tarantulas. **a**, **b** Anteriad load, *x* (red). **c**, **d** Dorsad load, *y*—muscle force (magenta), ventrad load—hemolymph pressure (blue; light blue in **d** values with low force/pressure ratios). **e**, **f** Proximad load, *z* (green). Compression: *S* < 0 if load > 0, e.g., anteriad/ventrad/proximad load; compression: *S* > 0 if load < 0, e.g., posteriad/dorsad/distad load. **a**, **c**, **e** *N* = 3; **b**, **d**, **f** *N* = 1. Regressions for mechanical sensitivity *S* [µε/mN] (*α* [°]): **a** *S*_ant_ = 12.77–7.44E−2 × *α*; **b** *S*_ant_ = − 8.45 + 3.98E−2 × *α*; **c** *S*_ven_ = 4.02–8.14E−3 × *α* (n.s.); *S*_dor_ = 3.35–6.33E−3 × *α* (n.s.); **d** see text, *S*_ven_ = 2.33 + 7.61E−4 × *α* (n.s.); *S*_dor_ = − 2.53 + 1.95E−4 × *α* (n.s.); **e** *S*_pro_ = − 1.33 + 8.22E−3 × *α*; **f** *S*_pro_ = 2.03–3.66E−2 × *α* + 1.39E−4 × *α*^2^; (tarantula; data: Blickhan and Barth 1985). Note the different scales

Any load to the metatarsus tip can be decomposed into its three components (Fig. 1): anteriad *x* (Fig. 2a, b), dorso-ventrad *y* (Fig. 2c, d), and proximad *z* (Fig. 2e, f). These components are classified as passive when load is picked up by the passive joint structures (anteriad *x* and proximad *z*), and as active when controlled by local muscles and hemolymph pressure (ventrad *y*). Being a dorsal hinge joint (Fig. 1), the tibia–metatarsus joint allows for the active control of the dorso-ventral loads.

*Passive loads* Turning first to the passive loads (1) directed anteriad (*x*) perpendicular to the plane of the tibia–metatarsus joint, and (2) pointed proximad (*z*) toward the joint, parallel to the metatarsus, it can be observed that the amount of strain strongly depended on joint angle, loading direction, and the site of measurement on the tibia. Strain depended linearly on force (force amplitude: ca. 25 mN; Blickhan 1985). The contributions of dorso-ventrad (*y*) loads induced by slow passive deflection were neglected (see below).

i. Anteriad loading results in anteriad bending of the tibia and torsion with respect to its long axis. Such loads induced positive strain at the site of the HS8 lyriform organ (*x*, Fig. 2a) with values about tenfold the values observed during proximad loading (comp. 2). Positive strain, i.e., slit dilation, cannot be detected by the sensory cells. Compression (strain < 0**,** which compresses the slits and stimulates the sensory cells supplying them) resulted from negative, i.e., posteriad loads. However, for the almost fully extended joint (joint angle *α* = 172°), strain at the HS8 vanished. Therefore, the organ may well be used to register torsion of the tibia, which increases with decreasing joint angle α, whereas at the same time, bending diminishes. Dorsally (Fig. 2b), the tibia was strained as well due to torsion but less than at the site of HS8.
ii. Depending on *α*, proximad load along the metatarsus generates load parallel to the long axis as well as dorso-ventral bending of the tibia. Compression (strain < 0) was observed for proximad loads and the flexed joint (*α* = 100°; *z*, Fig. 2e, f). Zero strain dorsally on the tibia was measured for a joint angle of 184°. There, the maximum compression was observed at 110°. At the site of the lyriform organ HS8, no strain was induced at 162°. During slow locomotion of *Cupiennius salei*, the tibia–metatarsus joint of the second leg was almost fully extended (*α* ≈ 170°) and the strain due to passive loads at the site of the lyriform organ HS8 minimized.

*Active loads* For dorso-ventrad loads at the distal metatarsus (Fig. 1) generated by muscles (flexion; ventrad load < 0) and hemolymph pressure (extension; ventrad loads > 0), the mechanical sensitivity *S* (*y*, Fig. 2c, d) varied considerably and its magnitude was independent of the joint angle *α*. Passive joint stiffness, i.e., the moment necessary to bend the leg in the ventrad direction without muscle activity or hemolymph pressure, was low enough for low deflection rates (ca. 0.15 °/s; joint stiffness 0.1 mN/°) to be neglected within the joint limits (Blickhan 1983, 1986). Correspondingly, the elicited strains were considered to be negligible. Passive deflections at higher rates (ca. 0.4 °/s and above) provoked muscle activity leading to strongly varying strain. The corresponding sensitivities were not documented in Blickhan (1983). The mechanical sensitivities for the active loads were determined from the peak values of strain and the force measured simultaneously at the tip of the metatarsus (variation between 2 and 20 mN; Blickhan and Barth 1985). The contributions of muscle force and hemolymph pressure were not separated in this study (see also note below). At the site of the lyriform organ HS8, muscular flexion (ventrad load < 0; see Fig. 1) led to compression (strain < 0) independent of joint angle. Hemolymph pressure (ventrad load > 0) caused similar tensile strain (strain > 0; *S*_m_ = 2.4 (1.6|3.0) µε/mN (median (25%|75%) percentile); *S*_P_ = 2.0 (1.9|4.2) µε/mN; *p* = 0.3, Wilcoxon, values, see Fig. 2c). As the mean mechanical sensitivity *S* was similar for both flexion and extension and independent of the joint angle *α*, the particular location of the lyriform organ (HS8) is well suited to register net torque generated actively at that joint. At the dorsal tibia (TiO), along its long axis, where intrinsic muscles attach, muscle contraction led to compression (*S*_m_ = 2.5 (2.1|2.8) µε/mN; Fig. 2d). This can be attributed to both the bending moments as well as to the axial load of the tibia due to the tension of the flexor muscles (see below). Surprisingly, hemolymph pressure (joint extension or ventrad load < 0) also generated compressive strain at this site [*S*_P_ = − 2.5 (− 2.2|− 2.9) µε/mN; absolute values *p* = 0.8, Wilcoxon].

The latter finding requires a closer inspection. The force/pressure ratios calculated from the time courses of force and hemolymph pressure (Blickhan and Barth 1985) are in general roughly as expected assuming that hemolymph pressure pushes against the whole cross-sectional area of the joint (see below). In contrast, in the documented measurements on the dorsal tibia (Blickhan and Barth 1985; Blickhan 1983), the ratios reach only 1/5th of the expected value. This finding explains itself by assuming that the hemolymph pressure was antagonized by simultaneous flexor muscle activity. Part of the *Musculus metatarsi longus* and the entire *M. met. bilobatus* attach at the inner wall of the dorsal tibia (Blickhan and Barth 1985). The vector of the resulting muscle force acting on the metatarsus points to the dorsal tibia. This dorsad component induces bending moments and compressional strain at the dorsal tibia, which in turn overrides the tension induced by hemolymph pressure. In fact, in a balanced situation, the resulting force approaches zero and mechanical sensitivities *S* can become infinitely large. By selecting instances with force peak readings, this problem was avoided. Nevertheless, the peaks measured, while the antagonists were active (low force/pressure ratio), should not be used to calculate mechanical sensitivities. Note: as muscles represent soft tissue, static pressure is transmitted into the muscle tissue. Internal muscle stress (*Cupiennius salei* ca. − 250 kPa; Siebert et al. 2010) is diminished by the counteracting hemolymph pressure (Karner 1999), i.e., muscle tension must exceed the pressure to be able to exert net forces to the skeleton. During leg flexion, the pinnate fibred flexors can generate high forces, but forces diminish if flexion causes a local increase in the quasi-static pressure. However, the latter will always be below muscle tension ("Appendix 2", Eq. 2). In our study, we measured the turning moment (force·lever arm) during extension. The turning moment generated by a known internal pressure at a joint can be calculated from geometry ("Appendix 3",   Eq. 3). The pressure is transmitted into the muscles independent of their respective activity. Thus, the effective area for leg extension is not reduced by active muscles running through this area as has been conceived by Blickhan and Barth (1985). The possibility to calculate the contribution of hemolymph pressure based on joint geometry strongly facilitates the consideration of the moments generated by hemolymph pressure.

## Reaction forces and strains in freely walking spiders (*Cupiennius salei*)

We here describe how the strains measured in freely running *Cupiennius salei* (Brüssel 1987; Barth 2002) can be expected both based on the mechanical sensitivities measured in tarantulas (Blickhan and Barth 1985; Barth 2002) and based on simple mechanical estimates. Furthermore, interpreting the strain measurements available, we here predict muscular flexion in the tibia–metatarsus joint, while *Cupiennius salei* is crawling upside down.

Strain in the spider exoskeleton results from both external and internal loads where torques caused by external loads can require balance by internal loads. During spider locomotion, the power stroke induced marked changes in the cuticular strain (Blickhan and Barth 1985; Barth 2002). The amplitudes of these strains were low as compared to the values observed in vertebrates and crabs (maximum measured values ca. 120 µε, tarantula, Blickhan and Barth 1985; ca. 300 µε, *Cupiennius salei*, Brüssel 1987, Barth 2002; vertebrates, typically 2500 µε, Biewener 1993; crab, ca. 3000 µε, Blickhan et al. 1993). We did not measure the strength of the cuticle. The estimates of modified primary Euler buckling strength (Currey 1967) point toward a high safety factor (ca. 10; "Appendix 4", Eq. 4) exceeding the values obtained for locusts (Schmitt et al. 2018) and vertebrates (Biewener 1993).

The loci of the lyriform organs did not stand out by being exposed to high strain during locomotion compared to the strain at several other sites at the dorsal and posterior tibia (Barth 2002; Blickhan and Barth 1985). For the second leg, the dependence of strain magnitude on walking speed was moderate but not significant (from 5 to 30 cm/s: mean strain magnitudes from − 10 to + 70% depending on the site (Barth 2002; Brüssel 1987). However, this may differ with leg number. Ground reaction forces of the first and third leg were more affected by the speed of locomotion (Weihmann 2007). The time course of the strains strongly depended on the site of measurement. Unfortunately, synchronous kinematic, dynamic, and strain recordings are only available for a few cases of freely walking spiders and lack a detailed documentation of the three-dimensional kinematics. Nevertheless, the following examples show how the strains in the spider exoskeleton provide information about biologically meaningful loads.

**Fig. 3:**
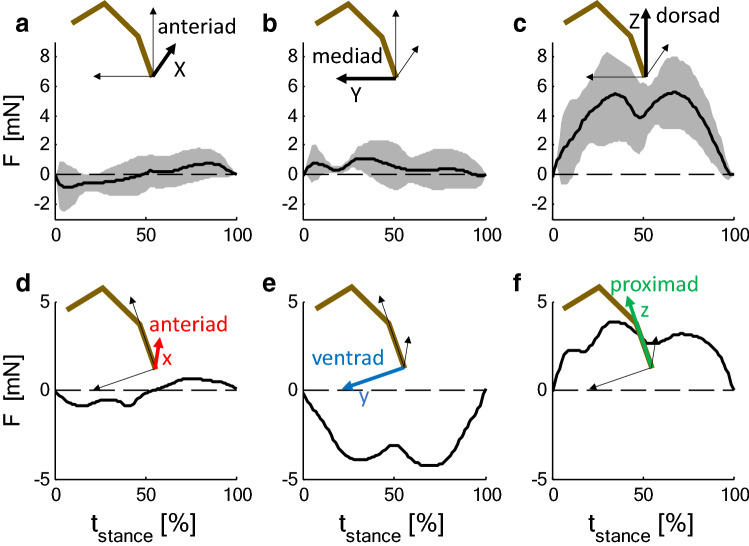
Time courses of the ground reaction force components *F* at the second leg during slow locomotion (10 cm/s) of *Cupiennius salei*. Forces ± SD (shaded; *N* = 4; *n* > 18) are normalized to a body mass of 3 g. **a**–**c** Components (*X* anteriad, *Y* mediad, *Z* dorsad) with respect to body orientation (global system; *X*, *Y*, *Z*). The reaction force has only minor anteriad and mediad components. **d**–**f** Components as calculated for a co-ordinate system (*x* anteriad, *y* ventrad, *z* proximad) with the *z*-axis parallel to the metatarsus and the *y*-axis within in the plain of the tibia–metatarsus joint (local system). Whereas the anteriad component is small due to the oblique position of the metatarsus, there is a considerable negative ventrad component which must be balanced by flexor activity at the tibia–metatarsus joint (Data: Brüssel 1987; Barth 2002)

**Fig. 4:**
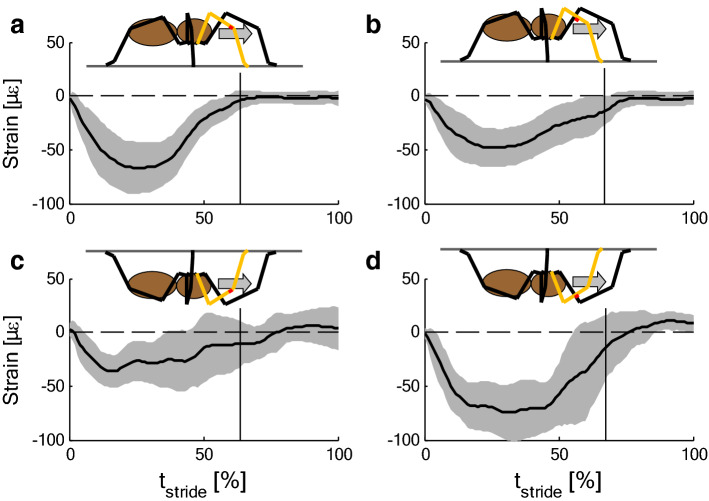
Strain (mean ± SD) during slow locomotion (5 cm/s) of *Cupiennius salei*. **a**, **b** Upside up (upright). **c**, **d** Upside down. Measurement sites: posterior distal tibia parallel the tibia axis (**a**, **c**), dorsal mid-tibia along the tibia axis (**b**, **d**). (**a** *N* = 4, *n* > 23; **b** *N* = 4, *n* > 17; **c, d** *N* = 3, *n* > 14). Data: Brüssel 1987. In inverted climbing, the footfall pattern and the strain varies strongly. Vertical lines indicate end of stance phase

During slow locomotion, the decomposition of the ground reaction forces (Fig. 3a–c) into a co-ordinate system parallel to the metatarsus (*Cupiennius salei,* Fig. 3d–f) revealed that anteriad force components almost vanished in contrast to proximad and ventrad forces. This was observed for all legs for *Cupiennius* and was supported for the fourth leg of the tarantula. During slow locomotion, Brüssel (1987) measured for *Cupiennius salei* a tibia–metatarsus angle of *α* = 170°, i.e., a value at which strain due to anteriad loading was small in tarantulas (Fig. 2). The ventrad force component requires activity of the flexor muscles in the tibia. Assuming a similar strain for a similar load relative to body mass (tarantula 13.2 g; hunting spider 3 g), according to the mechanical sensitivities (Fig. 2), a strain of about − 30 µε is to be expected both perpendicular to the slits of the lyriform organ HS8 and dorsally on the tibia in the direction of its long axis (anteriad||ventrad||proximad: HS8 (− 0.10||− 31.23||0.85) µε; dorsal mid-tibia (1.33||− 32.02||− 2.21) µε). This equals to about half the values actually measured both in the freely walking *Cupiennius* at the distal posterior and dorsal tibia (Fig. 4; data: Brüssel 1987). Strong deviations at the distal posterior tibia are expected due to the different site and orientation of the gauge. The magnitude and orientation of the strain may change strongly in the vicinity of the distal edge. At any rate, the flexor muscles balancing the weight of the animal cause compressive strain both at the site of the distal proximal tibia, and the dorsal tibia along the tibia long axis; this is supported by the mechanical sensitivities measured in the tethered animal.

**Fig. 5:**
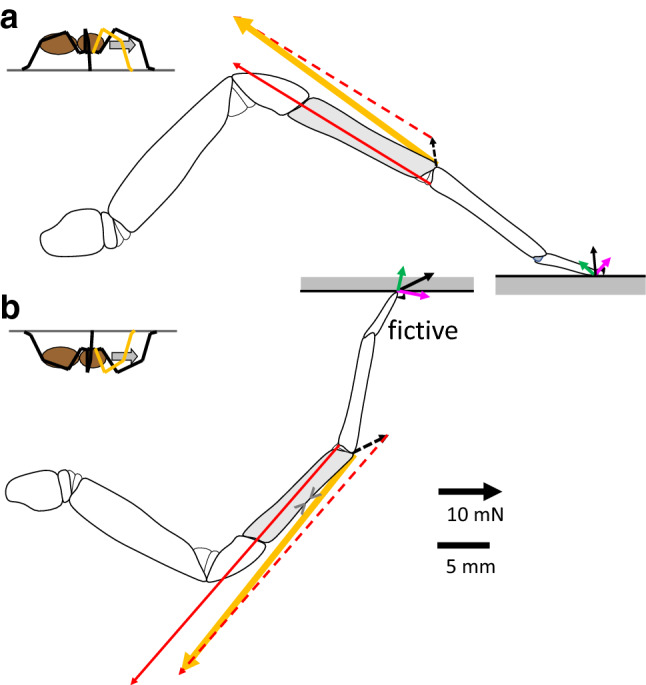
Loading of the tibia–metatarsus joint of the hunting spider *Cupiennius salei* **a** as reconstructed from the measurements of the ground reaction forces during slow locomotion (10 m/s; midstance; Fig. 4) upside up (upright) at level ground and **b** upside down with forces as assumed (fictive) to be necessary to generate compression at the dorsal tibia (>< ; Fig. 5). Forces: black—resultant vector of the ground reaction force (GRF); green: proximad component of GRF (in **b** negative, i.e., distad); magenta—dorsad component of GRF; red—muscle force (Fl. met. longus and Fl. met. acc.); yellow—resulting joint force. Dashed: vectors defining the resultant joint load (inertia and weight of the distal segment neglected); dashed black: resultant ground reaction force; dashed red: muscle force. Strain calculated based on ground reaction force and geometry: − 23.4 µε. For details see "Appendix 5", Eqs. (5, 6). **b** Calculated strain: − 22 µε

Another independent estimation of strain during locomotion is the following. Approximately, the tibia of a spider is a thin-walled tube with an obliquely cut end where the tibia–metatarsus joint is located. For known joint loads, we are able to calculate the deformation of the tube far from the joint. The measurement site on the dorsal tibia, used in the experiments on freely walking spiders (Brüssel 1987; Barth 2002), was located far from the joint. The force resulting from the measured ground reaction force at the dorsal hinge joint induces bending and compression of the dorsal tibia along the tibia axis (Fig. 5a). This was directly measured by strain gauges positioned dorsally on the tibia. (A gauge oriented perpendicular to the long axis would not register axial bending strain, but strain induced by buckling of the thin tibial tube.) With − 22.4 µε, the calculated strain (for details, see Fig. 5 and "Appendix 5") is lower than the value measured with the strain gauge (− 46 µε). To illustrate the sensitivity of the calculations regarding morphological details: a 25% reduction of diameter and wall thickness of the tibia would double the calculated compressional strain.

Surprisingly, in trials with animals climbing upside down underneath a cardboard, the strains measured during stance at the distal posterior and the dorsal tibia were both compressive (*Cupiennius salei,* Fig. 4c, d; Brüssel 1987). As compared to locomotion upside up (upright; Fig. 4a, b), compressional strain at the site of the HS8 was reduced, whereas it even increased at the dorsal tibia. Climbing upside down strongly affects the orientation of the ground reaction forces with respect to the legs. During upside up (upright) locomotion, the ground reaction forces push proximad to support body weight, but they pull distad while climbing upside down. Nevertheless, the measured compressions parallel to the long tibia axis at the distal proximal and the dorsal tibia are well explained provided the load of the tibia–metatarsus joint was dominated by the activity of the flexor muscles (Fig. 5b). This flexion generates an inward pull of the leg tip**,** which in turn, as compared to an outward push, doubles adhesion (Wolff and Gorb 2013) at the distal pads. Inward pulling to enhance adhesion and to improve attachment was also observed in treefrogs climbing on overhanging surfaces (Endlein et al. 2013), in climbing cockroaches (Larsen et al. 1995; Goldman et al. 2006), in geckoes (Autumn et al. 2006), and others (beetles, ants, and stick insects, etc.; recent rev.: Federle et al. 2019). Inward pulling forces were also found for the upslope leg of ants ascending or descending a slope of 60° (Wöhrl et al. 2017). A numerical model restricted to the sagittal plane predicts proximad pulling forces in the legs of insects for slope angles exceeding critical values above about 40° (Günther and Weihmann 2011, 2012). This includes climbing on ceilings. Consideration of attachment advantages may further improve the numerical predictions. In principle, secure attachment provided, a spider suspended on the cardboard would neither require activity of the flexor muscles nor hemolymph pressure. Even suspending the whole body with a single leg would induce a positive strain of about 3 µε only. However, the strain measurements prove that to prevent them from falling, the heavy spiders flex their legs. Ground reaction forces available for slow upside up (upright) locomotion (*Cupiennius*: Brüssel 1987; *Ancylometes*: Weihmann et al. 2012; *Aphonopelma* sp, *Brachypelma* sp., Blickhan and Barth 1985; Brüssel 1987) were converted to components in a metatarsus-fixed system of co-ordinates allowing the reconstruction of joint loads for two examples (*Cupiennius*, Brüssel 1987; tarantula, Blickhan and Barth 1985). From measurements on tarantulas standing in ascending position on a slope of 45° (Brüssel 1987; Barth 2002), it can be deduced that the second leg pulls in this posture demanding high flexor activity. For spiders crawling at the ceiling, no measurements of the ground reaction forces are available.

The tibia–metatarsus joint has only limited abilities to actively balance external torque in all directions. Force components of the reaction force perpendicular to the tibia–metatarsus plane (i.e., anteriad forces) can be controlled only to a minor extent by flexor muscles attaching at the anterior and posterior edge of the metatarsus (*M. fl. met. bilobatus*). In general, slender exoskeletons with hinge joints are vulnerable with respect to torques perpendicular to the joint axis. The calculations of the metatarsus-fixed components of the ground reaction force underline that an anteriad (*x*) force in this system of co-ordinates is avoided (Brüssel 1987; Blickhan and Barth 1985). Anteriad (*X*) forces with respect to the global system of co-ordinates fixed to the prosoma and oriented in the direction of movement are generated by placing the leg and adjusting the orientation of the plane of the leg as defined by the orientation of the major hinge joints. Avoidance of anteriad force components (*x*) in the local metatarsus-fixed system requires an adaptation of leg posture which is controlled at the coxa-trochanter joint. In consequence, high torques at the coxa-trochanter joint are expected with respect to an axis parallel to the major hinge joint axes (Dallmann et al. 2016; Zill et al. 2018).

Located at the distal end of the tibia, the lyriform organs (e.g., HS8) are sensitive to posteriad (i.e., negative anteriad) joint loading (e.g., Barth and Bohnenberger 1978). However, we have seen in the experiments on tethered animals that strain due to anteriad load was low for an almost extended joint. During slow locomotion, the tibia–metatarsus joint of the second legs was almost fully extended. Obviously, the animals used joint positions which avoided dangerous torques. In addition, the low lateral force reduces torsion as well as lateral bending. Small anteriad forces were observed for all legs during slow locomotion (*Cupiennius salei,* Brüssel 1987). Both, the almost extended tibia–metatarsus joint and the avoidance of lateral force ensure the intactness of the tibia–metatarsus joint. We have seen that muscular flexion induced considerable compression in the vicinity of the lyriform organ HS8 and we registered such compressions during slow locomotion in tarantulas and *Cupiennius salei*, despite of low anteriad force components (Blickhan and Barth 1985; Barth 2002). However, while running at high speed or while initiating a sprint or jump leg kinematics and the directions of the ground reaction, force vectors may strongly deviate from those found during slow locomotion.

Considering the dorsad load and its influence on tibia deformation, it is essential to recall that co-activation of antagonists strongly alters joint load and thus loading of the skeleton. According to our mechanical measurements (Fig. 2), the lyriform organ HS8 is able to register net torque with respect to the hinge axis of the joint. A balance of muscular torque by pressure-induced torque is suitable to cancel cuticular strain at the HS8. In freely walking hunting spiders, both tension and compressive strain were observed at higher speeds (30 cm/s) at the distal posterior tibia parallel to its long axis (Brüssel 1987). Sudden changes of sign during stance indicate variable anteriad forces and a flexed tibia–metatarsus joint rather than sudden leg extensions**,** which would hamper progression. In fact, at this rather high speed, the tibia–metatarsus joint flexed from 131° to 101° and the second leg sometimes took over the function of the first and sometimes the function of the third leg (Reinhardt 2006; Weihmann 2013).

The information from different lyriform organs in the region of the tibia–metatarsus joint could be combined by the spider central nervous system to differentiate between loads and loading direction (Blickhan and Barth 1985; Barth 2002). The mechanical sensitivities at the various sites indicate that muscles generated negative (i.e., adequately stimulating) strain at the site of the posterior (HS8, HS9) and anterior tibia (VS4) perpendicular to their slits. The ventral organ (VS5) was compressed by hemolymph pressure (dorsad load). Activity of the VS5 could be used to correct for the influence of pressure at the sites of the HS8, HS9, and VS4. Anteriad loads caused negative strain at the VS4 (at *α* < 150°), posteriad loads caused negative strain at the HS8 (*α* < 180°) and VS5 (*α* < 180°). Whereas muscles led to compression at the contralateral organs (HS8, HS9, VS4), anteriad and posteriad loads compressed the organs at the ipsilateral side only. The corresponding sensory information can in principle be used to distinguish between actively controlled dorsad loads and the passive anteriad loads.

## Evaluating the magnitude of strain

Knowing the exoskeletal strains and their sensory reception are both highly relevant for an in depth understanding of the biological significance and adaptive specialization of the spider extremities for locomotion. We therefore add a critical evaluation of the virtues and caveats of strain gauge measurements, which are demanding in regard to both practical technology and theoretical interpretation.

We focus on the magnitude of the strains actually measured and on the suitability of the strain gauge technology to indicate them precisely, taking the size of the investigated object, material properties, and recent interferometric observations on *Cupiennius salei* into account. The amplitudes of the strain fluctuations measured in the leg cuticle of hunting spiders and tarantulas typically ranged from 10 to 100 µε. During fast runs and starts, they reached up to 200 µε. The values obtained differed depending on the specific measurement site, gauge orientation, and the spider’s behavior (see above).

In the tibia of the second walking leg of *Cupiennius salei*, high compressive strains during level walking in the order of − 100 µε were measured on the dorsal tibia and on the posterior distal tibia. Strains were lower (ca. − 15 µε) at the posterior and anterior mid-tibia with a slight tendency to increase with the speed of locomotion (from 5 to 30 cm/s; Barth 2002). While climbing upward and downward, this pattern was maintained with higher values during climbing (dorsal mid-tibia: − 133.3 µε ± 24.9 SD). The highest compressional strain of − 375 µε was found at the distal posterior tibia during walking at a speed of 30 cm/s (Brüssel 1987).

**Fig. 6:**
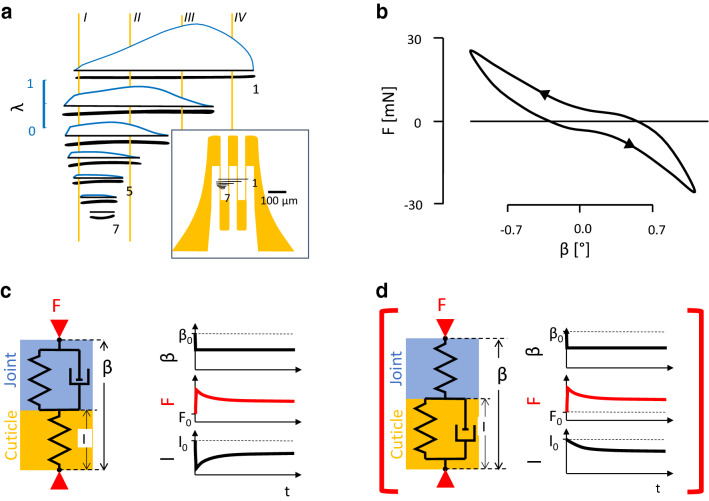
Factors affecting the strain registration and their impact in different setups. **a** Integration of deformation: a strain gauge covering the lyriform organ HS8 (right inset to scale) registers the sum of the slit deformation remaining after the attachment of the gauge divided by wire length. The inset depicts the selected strain gauge (yellow) covering the lyriform organ (black: lyriform organ HS8, 1. 7: slit numbers; to scale). In the main figure, the vertical distance between the slits (bold black horizontal lines) is expanded. Yellow vertical lines I…IV: wires of the gauge (comp. inset). Blue curves: deformation of the slits (*λ*) obtained by FEM and interferometry (*λ* = deformation of slit/(6.57 × 10^–4^ × length of slit) [m]; for details, see Hößl et al. 2009; Schaber et al. 2012). **b** Joint stiffness during cyclic triangular anteroposteriad deflection (upper arrow) at the metatarsus tip (tarantula; leg angle *α* = 160°, Fig. 1). *F* force; *β* deflection (anteriad: *β* > 0, for orientation see Fig. 1; 1 mm ≡ 3.5°; rate 0.1°/s; see material and methods). **c**, **d** Delineating visco-elastic properties using a standard linear solid. Viscosities can be found both at the joint (see **b**) and in the cuticle and the strain gauge. The examples describe extreme situations to identify the dominating viscosities. **c** The quick step deflection *β* at the leg tip causes an immediate rise in force. It compresses the cuticle spring (l) as the viscous element is rigid. While maintaining the deflection, the viscous element at the joint gives in. The two springs in series now in effect are less stiff than each single spring and the force drops. Correspondingly, the initial compression of the cuticle spring is reduced. This is the behavior observed in our measurements. **d** Alternative: the initial deflection compresses the joint and viscosities in the cuticle hamper its initial deformation. The joint is slowly compressed, while the external deflection is maintained. This is not observed (red brackets). *β*_0_, *F*_0_, *l*_0_: starting values

The spider’s leg segments represent thin-walled (typically *h*_c_ = 90 µm) tubes. A strain gauge bonded to its surface stiffens the cuticle and affects the stress distribution. For axial compression, Young’s modulus of the hydrated tibia of *Cupiennius salei* was measured as *E*_c_ = 18 ± 7 GPa (*n* = 16; *N* = 2; second leg, see below; Blickhan and Barth 1985). The strain reading of the gauge and the strain estimated from the externally visible compression of the whole probe (including dental cement at its ends) deviated less than 10%. Reducing the epoxy base thickness (by ca. 40%; *h*_b_ = 25 µm) of the gauge by grinding did not affect the strains registered at the site of the HS8-compound (lyriform) slit sense organ on the posterior aspect of the distal tibia (Figs. 1 and 2). Effectively, stiffening due to the strain gauge base and the adhesive reduce the strain by ca. 5% only. However, the constantan alloy foil making up the solder taps and the resistance wires of the gauge (Fig. 6) may strongly affect the stiffness of the gauge-cuticle composite. Ignoring the etching of the constantan foil, i.e., assuming that the gauge is completely covered with the constantan foil, a stiffening of the cuticle by 50% would be expected ("Appendix 6", Eq. 7). This certainly overpredicts the effect of the foil. Reversely, even the 10% increase in stiffness estimated experimentally during axial compression of the tibia could affect local stress distribution and thus strain magnitude. The example described above where strains induced during locomotion were calculated from ground reaction forces (Fig. 5 and "Appendix 6") confirms the validity of the order of magnitude of the strains actually measured. We also attached strain gauges to the meropodite of ghost crabs when investigating gait transitions (Blickhan et al. 1993). There, strain reached a value of about 3000 µε during jumping, despite the expected stiffening of the cuticle due to the application of the gauges by up to a factor of 5 (*h*_c_ = 220 µm; *E*_c_ = 0.6 GPa; Chen et al. 2008).

In tethered spiders (Fig. 1a), when loading the distal metatarsus tip sideways (anteriad, implying torsion and bending for the tibia), the mechanical sensitivity was *S* ≈ 1.6 µε/mN at the site of the lyriform organ HS8 at a joint angle *α* of 150° (tarantula; Blickhan and Barth 1985; see above). In interferometric measurements of slit deformation, following the same application of load in the hunting spider *Cupiennius salei* (Schaber et al. 2012), values of mechanical sensitivity of up to 47,800 µε/mN (4.78%/mN slit 1) were found for the slits of the same lyriform organ HS8. This difference, first of all, points to the difference between slit face deformation in untreated cuticle and the strain of a strain gauge covered area of cuticle (see below). It also stresses the outstanding mechanical sensitivity of this mechanoreceptor and the effective increase of its mechanical compliance due to its location, orientation, and micro-structure. Several additional factors contribute to the huge difference found by the two sets of experiments. As argued in the following, these are A *joint properties,* with *A*1 joint stiffness and *A*2 visco-elastic properties, and *B* effects of *strain gauge application,* with *B*1 local strain distribution, *B*2 stiffening, *B*3 buckling, and *B*4 adhesive filling.

A. The tibia–metatarsus joint transmits the load applied at the distal metatarsus to the cuticle in the environment of the organs. The stiffness of the joint in the tarantula is much higher than in *Cupiennius salei, *which explains part of the observed differences. The joint’s visco-elastic properties combined with different loading rates (in the strain measurement on tarantulas and the interferometric measurements on *Cupiennius salei*) do not explain the discrepancy between the two independent experiments as explained in the following.

*A1. Joint stiffness* Differences in joint stiffness strongly contribute to the differences in sensitivity. Torsional stiffness measured by applying posteriad deflection (joint angle *α* = 150°) of the joint region is much lower in the hunting spider *Cupiennius salei* (1.6 µN/°; Blickhan 1983; Schaber et al. 2012) than in tarantulas (37 µN/°; Blickhan 1986), where a roughly 23-fold load would lead to the same posteriad rotation. Assuming that the same angular rotation would result in a similar deformation for both species at the site of the slit sense organs. The far-field strain of 250 µε/mN extrapolated from the interferometric measurements in *Cupiennius salei* (see below) would correspond to a sensitivity of ca. 10 µε/mN in the tarantula, which is about sixfold the value obtained with the strain gauge.

*A2. Viscoelastic effects* Due to the different loading rates in the two sets of experiments, the visco-elastic properties of the joint diminished the difference between the sensitivity obtained with strain gauge technology and the interferometric measurement, respectively. Experiments with similar loading rate would have enhanced the difference, as the higher rate would induce a higher force and, as a result, a larger compression of the slit sense organ. To allow for interferometric data collection after the relaxation had leveled off**,** Schaber et al. (2012) used a very slow net deflection rate (Blickhan and Barth 1985: 0.4°/s; Schaber et al. 2012: 0.0065°/s). As seen in the deflection time curves, loading more than halved while waiting for 60 s after quick (0.13°/s) deflections. However, viscosities in the region of the organs being high as compared to joint viscosity could result in an increase in slit deformation for the same amount of deflection (Fig. 6d). In contrast, our strain gauge measurements rather supported a decrease in deformation (Fig. 6c). Increasing deflection rate led to increased strain. The effects of force relaxation due to joint viscosity are overriding any viscous effects at the site of strain measurement. The exponent *k* describing both the force and the strain relaxation (~ *t*^−*k*^) were found to be within 0.06 < *k* < 0.11 depending on method and subject. This represents a drop factor for strain of 0.7–0.45 within three powers of ten of time (tarantulas; 0.1 s ≤ time ≤ 100 s; Blickhan and Barth 1985; Blickhan 1986). The effects of viscosity only slightly reduce the overall compression of the cuticle at the lyriform organ’s location. In the interferometric experiments, fast deflection rates as used for the tarantula would have resulted in even higher strain values (factor 1.4–2.2).

B. The strain gauge is unable to resolve differences in strain below itself and its application per se affects the deformation of the corresponding cuticular area. The integration of deformations underneath by the gauge represents the strongest factor explaining the differences of the strain gauge measurement in the tarantula and the interferometric measurements in *Cupiennius salei*. Stiffening of the cuticle due to the strain gauge applied reduces the observed strain in the cuticle covered by it. The changes of local buckling due to the gauge application contribute to the observed differences, as well. Furthermore, when applying the gauge, the adhesive will reduce slit face deformation by filling the trough formed by its outer membrane, which is crucial for stimulus transmission to the sensory dendrite by its strain-induced deformation. These aspects are further discussed in the following:

*B1. Local strain distribution* Covering a strongly inhomogeneous strain field at the site of a lyriform organ, the strain gauge registers less than the mean of the strains. If we simplify and assume that deformation only occurs at the site of the slits, and take into account that the strain gauge integrates those deformations, then the strain registered would be in the order of 450 µε instead of local strains of 48,000 µε at the center of slit 1 (Fig. 6a; see also Blickhan and Barth 1985). Lyriform organs represent holes in the cuticle causing stress concentration and large local deformations. Following the results from FEA (finite-element analysis; Hößl et al. 2007, 2009), the strain of 450 µε at the site of the lyriform organ HS8 corresponds to a far-field strain (undisturbed by a hole) of ca. 250 µε.

*B2. Stiffening* Using the indentation method, Young’s moduli and hardness of puncture devices (chelicera, claws etc.) of *Cupiennius salei* were recently determined (Tadayon et al. 2020; for biomaterials: Labonte et al. 2017). The highest moduli measured in the chelicera reached about 20 GPa and are achieved by crosslinking and zinc-supplementation. At claw regions devoid of metal ions, the modulus was about one-third of our estimate (ca. 7 GPa versus 18 GPa). Based on this lower modulus, the stiffening due to the applied strain gauge would diminish the strain readings by a factor of up to 2.4 (no edging of the foil assumed, for calculation, see "Appendix 6", Eq. 7).

*B3. Local buckling* Heterogeneities in material properties and thickness caused buckling in the vicinity of the lyriform organ HS8 by load application (Schaber et al. 2012). Due to bending of the cuticle, local strain at the surface is amplified. An attached strain gauge enhances the total thickness of the bent structure with the outer constantan layer (solder tabs and measuring wires) having a higher Young’s modulus than the cuticle ("Appendix 7", Eq. 8). As a result, bending stiffness increases and the neutral plane of bending is moving outwards, from 0.5 to 0.9 *h*_c_ as measured from the base of the layers (h_c_: thickness of the cuticle). This in turn diminishes the distance between the neutral plane and the constantan layer (ca. 0.7 *h*_c_; total thickness cuticle, adhesive, base of the gauge and constantan layer: 1.67 *h*_c_). The combination of stiffening and the shift of the neutral plane reduces the strain by a factor of ca. 4.

*B4. Adhesive filling* The adhesive, filling the trough formed by its outer membrane, reduces the slit face deformation as measured in the interferometric experiments on naturally intact organs and their slits. As filling depth is only about 1/30th of cuticle thickness and is limited to the outer aspect of the slits, this stiffening most likely has only a minor effect on the far-field strain, but it reduces the estimated integrative strain (*B*1).

The influence of strain integration (*B*1) combined with differences in joint stiffness (*A*1) is the dominant factors explaining most of the seeming discrepancy between the slit deformations measured interferometrically and the strains measured applying strain gauge technology. More detailed information about structure and material properties in the cuticle and its local specializations would allow a more rigorous interpretation of the strain gauge measurements. In the future, a non-contact method such as speckle photometry could complement the interferometric investigations in setups with tethered animals. Here, deformation causes a change of the speckle patterns reflected from the illuminated surface. These changes registered with a camera can be used to measure strain fields (digital image correlation). However, this method is not suitable for experiments with a freely moving animal. Unfortunately, ultra-miniature strain gauges with reduced Young’s moduli, e.g., based on piezoelectric or piezoresistive polymers, are not available yet.

## Conclusion

Because of the small size and the thinness of the walls of arthropod skeletons, the measurements of cuticular strain using foil strain gauges are close to their physical limits. The reassessment of the studies available nevertheless demonstrates how strain measurements on spiders can be used to obtain basic information with respect to the biomechanics of locomotion and the role of natural strain detectors embedded in the skeleton. In future studies, especially more detailed information on the structure and material properties of the cuticle and their local specializations would allow improvement of strain gauge measurements by a more rigorous interpretation of the data received. At present, there is no alternative to the strain gauge method when it comes to the measurement of stresses and strains in the cuticle of freely moving animals.

## Data Availability

The review is based on data presented in cited publications.

## Appendix 1: Curved substrate

The increase in strain, $\Delta \varepsilon$, due to the measurement on a curved substrate is given by:

1 $$\Delta \varepsilon =\varepsilon \frac{{h}_{a}+{h}_{b} }{{r}_{Ti}}.$$

With adhesive thickness $h_{a} = 25\;{\upmu {\text{m}}}$, strain gauge foil base thickness $h_{b} = 25\;{\upmu {\text{m}}}$, radius of curvature (tibia radius), and ${r}_{Ti}=1 \text{mm}$, Eq. (1) gives $\frac{\Delta \varepsilon }{\varepsilon }=0.05$.

## Appendix 2: Pressure producible within a cylinder like closed compartment by muscle contraction

The pressure, $P$, developed by muscles within a pressurized compartment pulling at a piston is given by:

2 $$P= {\sigma }_{m}/\left(\frac{{A}_{P}}{\left({A}_{m}\bullet \xi \right)}+1\right).$$

With physiological cross section of muscle ${A}_{m}=4{ \text{mm}}^{2}$, cross-sectional area of piston ${A}_{P}=4 {\text{mm}}^{2}$, muscle tension ${\sigma }_{m}=250 \text{kPa}$ (Siebert et al. 2010), and the factor considering reduction of muscle force (e.g., due to pinnation) $\xi =1$, Eq. (2) gives a pressure of $P=125 \text{kPa}$.

## Appendix 3: Pressure generated joint torque

The torque, ${M}_{P}$, developed by hemolymph pressure, $P$, based on joint geometry (after Parry and Brown 1959; Blickhan and Barth 1985):

3 $${M}_{P}=P\bullet \frac{\text{d}V}{\text{d}\alpha }=P\bullet {A}_{P}\bullet r.$$

$\text{d}V$ displaced volume, $\text{d}\alpha$ rotation. With an effective area ${A}_{P}=4 {\text{mm}}^{2}$, a distance of the center of the effective area with respect to the dorsal hinge joint axis $r=1.4 \text{mm}$, and a pressure of $P=100 k\text{Pa}$, Eq. (3) gives ${M}_{P}=560 \text{mN}\, \text{mm}$.

## Appendix 4: Modified Euler buckling strain in the tibia of *Cupiennius salei*

Based on Currey (1967), the modified primary Euler buckling strain ${\varepsilon }_{\text{Euler},\text{mod}}$ can be given as:

4 $${\varepsilon }_{\text{Euler},\text{mod}}=\mathrm{0,12} \bullet {\left(\frac{\pi }{2{l}_{Ti}}\right)}^{2}\bullet \left({{r}_{Ti}}^{2}+{\left({r}_{Ti}-{h}_{c}\right)}^{2}\right).$$

With wall thickness $h_{c} = 90\;{\upmu {\text{m}}}$, tibia length ${l}_{Ti}=12 \,\,\text{m}$, and tibia radius ${r}_{Ti}=1 \text{mm}$, Eq. (4) gives ${\varepsilon }_{\mathrm{Euler},\mathrm{mod}}=3760 \;{\upmu}\upvarepsilon$.

## Appendix 5: Strain in dorsal tibia of *Cupiennius salei* during slow walking (diagram see Fig. 5)

The ventrad component of the ground reaction force, ${F}_{\text{ven}}$, is balanced by the force, ${F}_{m}=31 \text{mN}$, of the flexor muscles (${F}_{\text{ven}}=-3.1 \text{mN}$; distance tibia–metatarsus joint–ground reaction force: $17 \text{mm}$; lever for flexors: $1.7 \text{mm}$; ${F}_{\text{pro}}=2.9 \text{mN}$). Neglecting weight and inertia of the metatarsus, the vector of the force, ${\overrightarrow{F}}_{\text{joi}}$, at the tibia–metatarus joint is given by ${\overrightarrow{F}}_{\text{joi}}={\overrightarrow{F}}_{\text{ven}}+{\overrightarrow{F}}_{\text{pro}}+{\overrightarrow{F}}_{m}$. The joint force generates a bending moment, $M$, at mid-tibia. The component of the joint force perpendicular to the long tibia axis causes a moment of $M=59 \text{mN}\, \text{mm}$, the force component parallel to the joint axis due to eccentric loading at the dorsal hinge joint $M=34 \text{mN}\, \text{mm}$. The strain, $\varepsilon$, exerted by the bending moment is given as:

5 $$\varepsilon =M\bullet {r}_{Ti}/\left({J}_{Ti}\bullet {E}_{c}\right),$$

with the second moment of inertia, ${J}_{Ti}$:

6 $${J}_{Ti}=\frac{\pi }{4}\bullet \left({{r}_{Ti}}^{2}-{\left({r}_{Ti}-{h}_{c}\right)}^{2}\right)\bullet \left({{r}_{Ti}}^{2}+{\left({r}_{Ti}-{h}_{c}\right)}^{2}\right).$$

With ${E}_{c}=18\,\text{ GPa}$, $h_{c} = 90\;{\upmu {\text{m}}}$, and ${r}_{Ti}=1\,\, \text{mm}$, both moments combined generate a strain of $\varepsilon = - 19\;{\upmu}{\upvarepsilon}$. The axial compression of the tibia exerted by the parallel component of the joint force contributes another $\varepsilon =-3.4 \;\upmu\upvarepsilon$.

## Appendix 6: Stiffening of the cuticle due to attached strain gauge

Attachment of the strain gauge to the cuticle stiffens the material and proportionally reduces the strain detected (Technical Information Micro-Measurements):

7 $$\text{stiffening}=\left({E}_{c}\bullet {h}_{c}+{E}_{a}\bullet {h}_{a}+{E}_{b}\bullet {h}_{b}+{E}_{f}\bullet {h}_{f}\right)/\left({E}_{c}\bullet {h}_{c}\right).$$

With adhesive ${E}_{a}=3.4\,\, \text{kPa}$ and ${h}_{a}=25 \;\upmu \text{m}$, gauge base ${E}_{b}=3 \;\text{GPa}$ and ${h}_{b}=25 \; \upmu \text{m}$, cuticle ${E}_{c}=18 \,\,\text{GPa}$ and ${h}_{c}=90 \;\upmu \text{m}$, constantan foil (no etching) ${E}_{f}=1.60\,\, \text{GPa}$, and ${h}_{f}=5 \;\upmu \text{m}$, Eq. 7 gives $\text{stiffening}=1.5$.

## Appendix 7: Shift of neutral bending axis due to stiffening

The absolute value of the moments exerted by the stresses during bending above and below the neutral axis balance each other. Assuming a linear deformation across all layers (Euler bending), this results in the condition that:

8 $$\sum_{i}\left({E}_{i} {\int }_{{e}_{i}}^{{a}_{i}}{(r-{r}_{0})}^{2}\text{d}r\right)=\sum_{j}\left({E}_{j} {\int }_{{e}_{j}}^{{a}_{j}}{(r-{r}_{0})}^{2}\text{d}r\right)$$

for the layers ($i$) above and below ($j$) the neutral axis, respectively. ${E}_{i,j}$ is Young’s modulus of layer $i,j$ (see Appendix 6; no etching of the constantan foil), ${a}_{i,j}$, ${e}_{i,j}$ upper and lower perpendicular distance from the basis, $r$ perpendicular distance from bottom of layers, ${r}_{0}$ distance of the neutral axis. The resulting cubic equation with respect to ${r}_{0}$ was solved with Matlab^®^ (MathWorks, Natick, MA, USA).

## List of symbols

A: Area
a: Upper distance of layer to neutral axis
COM: Center of mass
COP: Center of pressure
*D*: Segment diameter
*d*: Derivative
*e*: Lower distance of layer to neutral axis
*E*: Young’s modulus
*F*: Force
Fl: Flexor
FEM: Finite-element method
GRF: Ground reaction force
*h*: Material thickness
*l*: Length
*J*: Second moment of inertia
*k*: Exponent describing stress relaxation
*M*: Moment, torque
MeTa, met: Metatarsus
*N*: Number of individuals
*n*: Total number of trials
*P*: Pressure
*r*: Radius, perpendicular distance from origin of layers (buckling, curvature)
S: Mechanical sensitivity (strain/load)
Sl: Slenderness
*t*: Time
Ta: Tarsus
Ti: Tibia
*V*: Volume
*x*, *y*, *z*: Local system of co-ordinates (Fig. 1)
*X*, *Y*, *Z*: Global system of co-ordinates
*α*: Tibia metatarsus angle
*β*: Anteriad deflection angle
Δ: Difference
*ε*: Unit for strain (dimensionless)
*λ*: Relative deformation of slits
*σ*: Stress
*ξ*: Pinnation factor

## Suffixes

0: Starting value (*t* = 0)
a: Adhesive
ant: Anterior, anteriad
b: Base (epoxid and adhesive)
c: Cuticle
f: Foil (constantan alloy)
i: Index of layer in the gauge application
j: Index of layer in the gauge application
joi: Joint
m: Muscle
dis: Distal, distad
P: Pressure
pos: Posterior, posteriad
pro: Proximal, proximad
ven: Ventral, ventrad
